# Memory-Like Responses of Brain Microglia Are Controlled by Developmental State and Pathogen Dose

**DOI:** 10.3389/fimmu.2020.546415

**Published:** 2020-09-25

**Authors:** Trim Lajqi, Milan Stojiljkovic, David L. Williams, Hannes Hudalla, Michael Bauer, Otto W. Witte, Reinhard Wetzker, Reinhard Bauer, Christian Schmeer

**Affiliations:** ^1^Institute of Molecular Cell Biology, Jena University Hospital, Jena, Germany; ^2^Department of Neonatology, Heidelberg University Children's Hospital, Heidelberg, Germany; ^3^Hans-Berger Department of Neurology, Jena University Hospital, Jena, Germany; ^4^Department of Surgery and Center of Excellence in Inflammation, Infectious Disease and Immunity, Quillen College of Medicine, East Tennessee State University, Johnson City, TN, United States; ^5^Department of Anesthesiology and Intensive Care Medicine, Jena University Hospital, Jena, Germany; ^6^Jena Center for Healthy Aging, Jena University Hospital, Jena, Germany

**Keywords:** microglia, LPS, β-glucan, trained immunity, tolerance, maturation

## Abstract

Microglia, the innate immune cells of the central nervous system, feature adaptive immune memory with implications for brain homeostasis and pathologies. However, factors involved in the emergence and regulation of these opposing responses in microglia have not been fully addressed. Recently, we showed that microglia from the newborn brain display features of trained immunity and immune tolerance after repeated contact with pathogens in a dose-dependent manner. Here, we evaluate the impact of developmental stage on adaptive immune responses of brain microglia after repeated challenge with ultra-low (1 fg/ml) and high (100 ng/ml) doses of the endotoxin LPS *in vitro*. We find that priming of naïve microglia derived from newborn but not mature and aged murine brain with ultra-low LPS significantly increased levels of pro-inflammatory mediators TNF-α, IL-6, IL-1β, MMP-9, and iNOS as well as neurotrophic factors indicating induction of trained immunity (*p* < 0.05). In contrast, stimulation with high doses of LPS led to a robust downregulation of pro-inflammatory cytokines and iNOS independent of the developmental state, indicating induced immune tolerance. Furthermore, high-dose priming with LPS upregulated anti-inflammatory mediators IL-10, Arg-1, TGF- β, MSR1, and IL-4 in newborn microglia (*p* < 0.05). Our data indicate pronounced plasticity of the immune response of neonate microglia compared with microglia derived from mature and aged mouse brain. Induced trained immunity after priming with ultra-low LPS doses may be responsible for enhanced neuro-inflammatory susceptibility of immature brain. In contrast, the immunosuppressed phenotype following high-dose LPS priming might be prone to attenuate excessive damage after recurrent systemic inflammation.

## Introduction

Microglia are the resident macrophage population that control the patterning and wiring of the brain in early development and contribute to homeostasis throughout life (1–4). Furthermore, microglia implement innate immunity in the central nervous system (CNS) as a first-line defense against invading pathogens by continuous microenvironmental surveillance (1, 5–9).

Alterations in brain homeostasis and by most pathologic events induce activation of microglia (10). During the activation process, microglial cells display specific adaptive functions, including migration toward injury, phagocytosis, antigen presentation, and synapse remodeling (11–13). Importantly, there is growing evidence indicating that microglial cells may lose the ability to retransform to a completely naïve status after activation and may remain as “postactivated” or “primed” microglia, exhibiting potential neuropathological relevance (2). In this connection, it has been demonstrated that peripheral inflammatory challenges in adult mice induce long-term alterations in microglial response, exhibiting either enhanced or suppressed immune functions possibly exacerbating or alleviating brain pathology in mouse models (14, 15). These results clearly indicate microglial capacity to develop innate immune memory (IIM), that is, long-lasting molecular reprogramming leading to either enhanced (trained immunity) or suppressed (immune tolerance) microglial responses to a delayed, secondary insult (16). In agreement with previous studies on monocytes [reviewed in (17)], we recently showed that opposite immune responses of IIM can be induced in microglia as a function of pathogen dose, specifically revealed for the endotoxin LPS (14, 15, 18).

Our present report extends the investigation of dose-dependent induction of IIM by evaluating the role of maturation on microglial memory response. Primary microglial cells from neonatal, adult, and aged mice were primed with ultra-low and high doses of LPS, and inflammatory responses to a secondary insult with LPS were assayed after 6 days in culture. Our data reveal trained immunity in response to ultra-low priming doses of LPS only in newborn microglia, whereas immune tolerance was induced in microglia in an age-independent manner (i.e., in naïve, mature, and aged microglia). The implications of age- and dose-dependent priming effects for microglial functional patterns in the CNS are discussed.

## Materials and Methods

### Animals and Microglia Isolation Procedures

Neonatal (P0–P3, *n* = 261), young adult (3 months old, *n* = 58), and aged (24 months old, *n* = 36) mice from a C57Bl/6J locally inbred mouse strain were used to isolate primary microglial cells. All experiments were carried out according to the guidelines from Directive 2010/63/EU of the European Parliament on the protection of animals used for scientific purposes and approved by the local authorities for animal welfare (permission numbers for tissue and organ harvesting: twz 07-2017, twz 31-2017, and twz 13-2020). Adult and aged C57BL/6J mice housed for breeding and organ harvesting were kept at 12-h light and dark cycles with free access to food and water.

Neonatal primary microglial cells were isolated from the cerebral cortex of newborn mice (6–12 newborn male and female mice brains were pooled, respectively) as described previously (18, 19). Briefly, newborn mice were decapitated, and heads were transferred into Petri dishes filled with ice-cold phosphate-buffered saline (PBS). Using fine scissors, the scalp was opened carefully along the midline, and the brain was removed. Then, meninges were removed, and cortices and hippocampi were collected in 15-ml tubes filled with PBS. Collected brains were processed in 2 ml dissociation media containing 200 μl 2.5% trypsin and further supplemented with 20 μl of DNAse I in order to digest DNA released from dead cells. After incubation at 37° C and 5% CO_2_ for 30 min, the medium was removed, and the brain tissues were suspended in 2 ml of Dulbecco's modified Eagle's medium (DMEM, SIGMA-Aldrich #06429, endotoxin tested) containing 10% heat-inactivated fetal bovine serum (FBS, SIGMA-Aldrich #F7524, endotoxin-free and sterile-filtered), 1% penicillin/streptomycin, 1% amphotericin B, supplemented with 30 μl DNAse I. Brain tissues were then homogenized and further transferred to T75 cell culture flasks with additional 8 ml culture medium and incubated at 37° C and 5% CO_2_ for 7 days, followed by medium change and further incubation for 7 more days.

Adult and aged male mice were sacrificed by an overdose of isoflurane, and brains were carefully removed after transcardial perfusion with ice-cold PBS for 5 min as described previously (20). Briefly, whole brains from 1–2 mice were harvested, meninges were removed, and brains were minced finely with a scalpel in Petri dishes containing enzymatic solution as described (21). After incubation at 37° C and 5% CO_2_ for 90 min, the enzymatic reaction was stopped by adding Hank's balanced salt solution (HBSS, SIGMA-Aldrich #H6648) supplemented with 20% FBS. The tissue was centrifuged for 10 min at room temperature, and the pellet was resuspended in HBSS containing 2 ml of 0.5 mg/mL DNAse I for 5 min. The brain tissue was homogenized and filtered through a 70-μm cell strainer. The collected cell suspension was centrifuged for 10 min followed by a gradient density separation using Percoll (GE Healthcare, #17-5445-02). The pellet that contained mixed glial cells was washed with HBSS and resuspended in culture medium supplemented with 5 ng/ml recombinant mouse granulocyte and macrophage colony stimulating factor (GM-CSF) (BioLegend, #576302). Suspended glial cells were placed in T75 cell culture flasks coated with poly-L-lysine and incubated at 37° C and 5% CO_2_ for 14 days. Medium was changed continuously until confluence was reached on day 14.

After 14 days, adherent microglial cells were separated from astrocytes by adding PBS-EDTA solution and careful shaking. After harvesting, microglial cells were seeded (75,000 cells/well) in adherent well plates. Long-term cultures of adult microglia were performed as previously described (20, 21). Purity of microglia was always in the range of 95–98% as confirmed by specific Iba1 staining (data not shown).

### Microglia Stimulation

Three different stimulation protocols were performed: (i) Microglia were stimulated twice following a two-step (“two-hit”) protocol as described elsewhere (14, 18). Microglia were initially stimulated (“primed”) with different doses of LPS (“first hit,” 1 fg/ml or 100 ng/ml for 24 h, respectively; *E. coli* serotype 055:B5 obtained from Sigma-Aldrich, St. Louis, USA) or β-glucan (100 fg/ml or 1 μg/ml for 24 h). Cells were restimulated 5 days after the first challenge (day 6) by a fixed dose of LPS (“second hit,” 100 ng/ml). Newborn, mature, and aged microglial cells were divided into 4 groups: Microglia of the first group were used unstimulated (*US Group*). The second group included unprimed microglia (*UP Group* without the first hit on day 1 but stimulated on day 6 with a fixed dose of LPS, 100 ng/ml). The third group (*ULP group*) included microglia stimulated with an ultra-low (1 fg/ml LPS or 100 fg/ml β-glucan) dose of stressors on day 1 and restimulated at day 6 with a fixed dose of LPS (100 ng/ml). The fourth group, labeled as the high-dose-primed group (*HP group*), was stimulated with a high dose (100 ng/ml LPS or 1 μg/ml β-glucan) of stressors and restimulated at day 6 with a fixed dose of LPS (100 ng/ml). In addition, microglia isolated from newborn and adult mice (ii) were primed by ultra-low (ULP, 1 fg/ml) or high (HP, 100 ng/ml) doses of LPS repetitively once a day for 3 days (repetitive (REP) group) or one time for 3 days (long-term [LT] group) followed by stimulation with a fixed dose of LPS (second hit, 100 ng/ml) on day 6 after priming (Supplementary Figure 1) or (iii) stimulated by ultra-low (ULP, 1 fg/ml) or high (HP, 100 ng/ml) doses of LPS repetitively once a day for 3 days (repetitive (REP) group) or one time for 3 days (LT group) without subsequent stimulation (Supplementary Figure 2).

**Figure 1:**
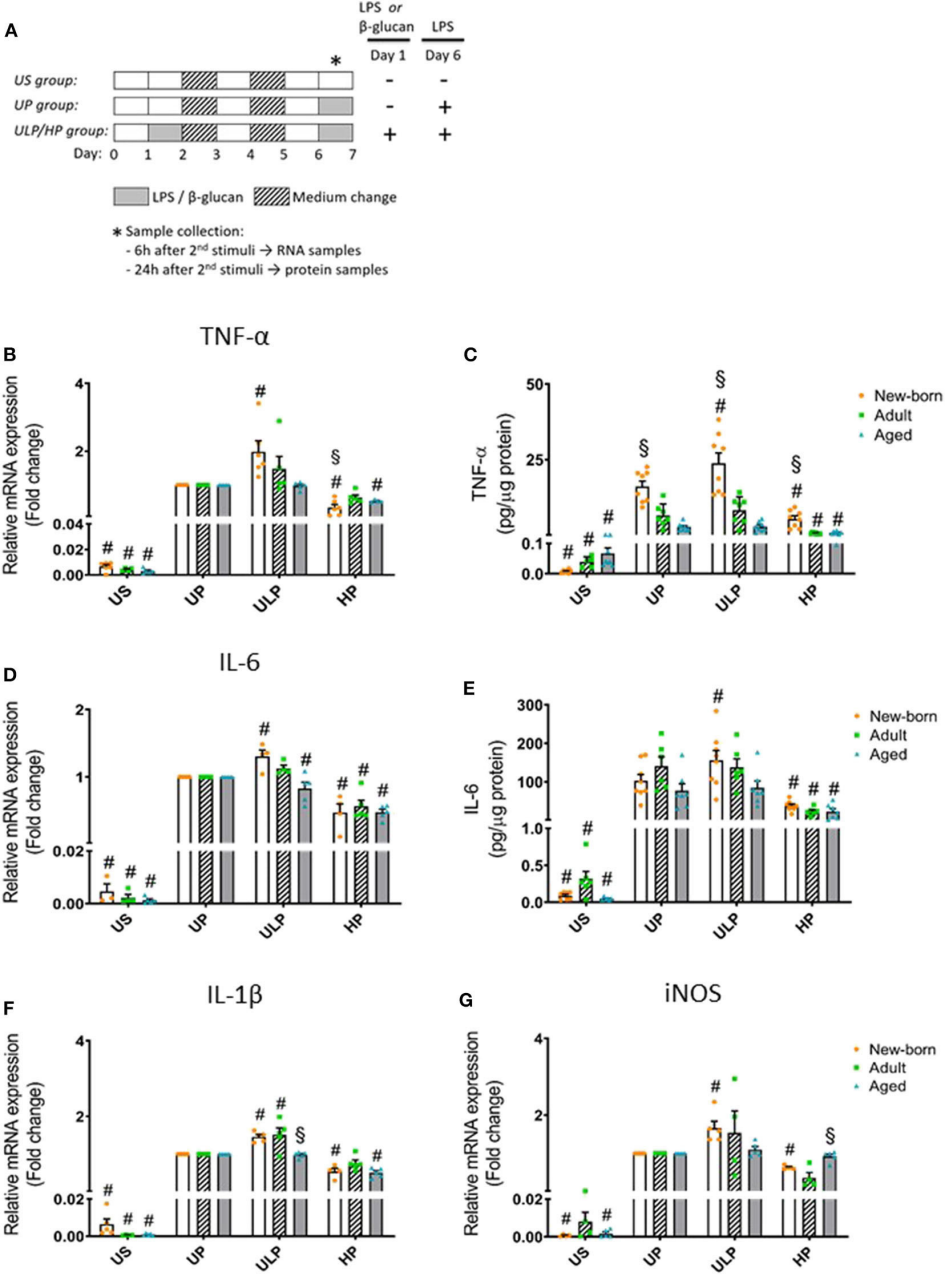
Effects of maturation and priming with low and high LPS dosages on pro-inflammatory responses of murine brain microglia. Microglia isolated from newborn (orange dots, open columns), adult (green dots, hatched columns), and aged (blue dots, gray columns) mice were primed initially by ultra-low (ULP, 1 fg/ml) or high (HP, 100 ng/ml) doses of LPS, followed by a second stimulation (day 6) with 100 ng/ml LPS **(A)**. The data were normalized and compared to unprimed microglia (UP Group without any stimulation at day 1 with stimulation at day 6 with fixed dose of LPS 100 ng/ml). Unstimulated microglia served as negative control (US Group without LPS stimulation neither at day 1 nor at day 6). RNA samples (6 h) and supernatants (24 h) were collected after the 2nd stimulation and analyzed for gene expression of TNF-α (* indicates time point of sample collection). **(B)** (*n* = 5–6), IL-6 **(D)** (*n* = 4–5), IL-1β **(F)** (*n* = 5), and iNOS **(G)** (*n* = 4–5) (unprimed cells assigned as 1.0) and cytokine production of TNF-α **(C)** (*n* = 7–8) and IL-6 **(E)** (*n* = 6–8) by ELISA (normalized to total protein concentration). Data shown in scatter dot plots represent means + SEM, ^#^*p* < 0.05 vs. unprimed conditions within each age group (naïve or mature), ^§^*p* < 0.05 vs. adult microglia within each stimulation condition (US, UP, ULP, HP).

To evaluate the effects of repeated stimulation with LPS, we normalized the data to the UP group (in the case of mRNA expression). The US group served as a negative control. Schematic overviews are shown in Figure 1A, and Supplementary Figures 1A, 2A.

### Cell Lysis and Protein Extraction

Cells were lysed using ice-cold RIPA buffer (containing 50 mM Tris/HCl pH 8.0; 150 mM NaCl, 1% (v/v) NP-40, 0.5% (v/v) Na-deoxycholate, 0.1% (w/v) SDS) supplemented freshly with a protease and phosphatase inhibitor cocktail (100 mg/ml Pefa-Block, 1 mg/ml Pep-statin A, 10 mM sodium vanadate, and 1 mg/ml Leupeptin). Samples were centrifuged (13,500 × g for 30 min at 4° C), and supernatants were mixed with 5x protein sample buffer (5% SDS, 33% glycerol, 25% β-mercaptoethanol, and 0.1 mg/ml bromophenol blue) and heated for 5 min at 95° C. Samples were used for normalization of cytokine amounts and lactate production levels.

### Measurement of the Protein Concentration and Analysis of Cell Viability by MTT Assay

Total protein concentration was determined using the Pierce™ 660 nm Protein Assay Kit (#22662) from Thermo Fisher Scientific (MA, USA). Ionic detergent compatibility reagent (IDCR) (#22663, Thermo Fischer Scientific) was used in order to reduce interference. Briefly, 10 μl of standard, sample, and blank in duplicates were plated in a 96-well plate, followed by immediate addition of 150 μl assay reagent supplemented with IDCR. Then, the plate was covered and shaken for 1 min in a plate shaker. Afterwards, the plate was incubated for additional 5 min at room temperature without shaking. Absorbance was measured at 660 nm using a TECAN Infinite 200 Plate reader (Tecan, Switzerland). Protein concentration was then calculated based on the values of the standard curve (Supplementary Figure 3).

Cell viability was determined using the MTT assay. Cells were seeded into a 96-well plate and incubated at 37° C (5% CO_2_) for 24 h. After attachment, primary microglial cells were treated according to the stimulation scheme depicted in Figure 1A. Twenty-four hours after the second LPS stimulation, MTT (3-(4,5-dimethylthiazol-2-yl)-2,5-diphenyltetrazolium bromide) solution was added and incubated for 4 h at 37° C (5% CO_2_). Next, the solubilization solution was added and incubated overnight at 37° C (5% CO_2_) for 24 h. Absorbance was measured at 570 nm using a TECAN Infinite 200 Plate Reader (Tecan, Switzerland), and data were shown as relative viability (UP assigned as 100%) (Supplementary Figure 3A).

### Cytokine Determination

Cytokine levels in supernatants were measured 24 h after final stimulation (14) using enzyme-linked immunosorbent assay (ELISA) kits for TNF-α (#430902, at a ratio of 1:40 except US at 1:1), IL-6 (#431302, at a ratio of 1:140 except US at 1:1), and IL-10 (#431412, at a ratio of 1:1) obtained from BioLegend (San Diego, CA), following manufacturer's instructions. Briefly, the 96-well plate was sealed and incubated with capture antibody (100 μl/well) overnight at 4° C. The plate was washed 4 times with washing buffer (0.05% Tween-20 in 1x PBS) and blocked by adding 200 μl/well assay diluent (1% BSA) in order to reduce the background and block non-specific binding. The plate was then sealed and incubated for 1 h at room temperature in a plate shaker (500 rpm). The plate was washed (4x) again, and 100 μl/well of prepared standards, diluted samples, and blanks were added. The plate was sealed and incubated for 2 h at room temperature with shaking. After 2 h, the plate was washed again (4x) and incubated with 100 μl/well detection antibody for 1 h at room temperature with shaking. The plate was washed again (4x) and incubated with 100 μl/well avidin-HRP for 30 min with shaking under dark conditions (light sensitive). Finally, after 30 min, the plate was washed (5x), and 100 μl/well of TMB substrate solution was added. The plate was incubated in the dark for 15–30 min, and the reaction was stopped by adding 100 μl/well of the stop solution (2N H_2_SO_4_) to the wells. Absorbance was determined with a VersaMax Microplate Reader (Molecular Devices, USA) at 450 nm and a second reference wavelength at 570 nm. Levels of TNF-α, IL-6, and IL-10 were normalized against the protein concentrations of each sample and depicted as pg/μg of total protein.

### Real-Time qPCR

Real-time qPCR was performed 6 h after final stimulation (14). To determine gene-expression levels, total RNA was extracted using QIAzol Lysis Reagent (#79306) purchased from Qiagen (Hilden, Germany). RNA concentration and quality were checked by using a Nanodrop ND-1000 machine (Peqlab, Erlangen, Germany). cDNA was synthesized using a RevertAid First Strand cDNA Synthesis kit (#K1612) from Thermo Fisher Scientific (Waltham, MA, USA). qPCR reaction was performed by using a LightCycler 480 SYBR Green (Roche, Switzerland) and Rotor gene Q machine (Qiagen, Germany). Primers used in the study are listed in Table 1. Housekeeping genes, GAPDH and HMBS, were used for normalization. Relative gene expression was calculated by the comparative C_T_ ($2_{\text{T}}^{-\Delta \Delta \text{C}}$) method (22).

**Table 1:** Primers used in the study.

**Gene name**		**Primer Sequences (5′- 3′)**
***Tnf-a***	Fw	CTGTAGCCCACGTCGTAGC
Tumor necrosis factor-a	Rev	TTGAGATCCATGCCGTTG
***Il-6***	Fw	CCTCTCTGCAAGAGACTTCCATCCA
Interleukin-6	Rev	GGCCGTGGTTGTCACCAGCA
***iNOS***	Fw	AAGGCCACATCGGATTTCAC
Inducible nitric oxide synthase	Rev	GATGGACCCCAAGCAATACTT
***Il-1β***	Fw	GGCAGGCAGTATCACTCATT
Interleukin 1 β	Rev	AAGGTGCTCATGTCCTCAT
***MMP-9***	Fw	ACCACTAAAGGTCGCTCGGATGG
Matrix metallopeptidase 9	Rev	AGTACTGCTTGCCCAGGAAGACG
***Il-10***	Fw	ACCAGCTGGACAACATACTGC
Interleukin 10	Rev	TCACTCTTCACCTGCTCCACT
***Il-4***	Fw	TGGGTCTCAACCCCCAGCTAGT
Interleukin 4	Rev	TGCATGGCGTCCCTTCTCCTGT
***Arg-1***	Fw	TCACCTGAGCTTTGATGTCG
Arginase-1	Rev	CTGAAAGGAGCCCTGTCTTG
***TGF**- β*	Fw	TGCTTCAGCTCCACAGAGAA
Transforming growth factor β	Rev	TACTGTGTGTCCAGGCTCCA
***Msr1***	Fw	TGGAGGAGAGAATCGAAAGCA
Macrophage scavenger receptor 1	Rev	CTGGACTGACGAAATCAAGGAA
***Bdnf***	Fw	GACAGTATTAGCGAGTGGGTCA
Brain derived neurotrophic factor	Rev	CCTTTGGATACCGGGACTTT
***Pfkfb3***	Fw	GGAGAGGTCAGAGGATGCAAA
6-phosphofructo-2-kinase/fructose-2,6-biphosphatase 3	Rev	GCTGTTGATGCGAGGCTTTT
***Tlr4***	Fw	ACCTGGCTGGTTTACACGTC
Toll-like receptor 4	Rev	CAGGCTGTTTGTTCCCAAAT
***MyD88***	Fw	TCCGGCAACTAGAACAGACAGACT
Myeloid differentiation primary response 88	Rev	GCGGCGACACCTTTTCTCAAT
***Irak4***	Fw	GTCATGACCAGCCGAATCGTG
Interleukin−1 receptor-associated kinase 4	Rev	CAGACACTGGTCAGCAGCAGA
***p65***	Fw	CTTCCTCAGCCATGGTACCTCT
Transcription factor p65	Rev	CAAGTCTTCATCAGCATCAAACTG
***Gapdh***	Fw	CATGGCCTTCCGTGTTTCCTA
Glyceraldehyde 3-phosphate dehydrogenase	Rev	CCTGCTTCACCACCTTCTTGAT
***Hmbs***	Fw	GTTGGAATCACTGCCCGTAA
Hydroxymethylbilane synthase	Rev	GGATGTTCTTGGCTCCTTTG

### Measurement of Reactive Oxygen Species (ROS)

ROS were measured using the H_2_DCFDA assay. The assay is based on the use of 2′,7′-dichlordihydrofluorescein-diacetat (H_2_DCFDA; #D399, Thermo Fisher Scientific, Waltham, MA, USA), a membrane-permeable reduced form of fluorescein, which reacts with reactive oxygen species, thereby emitting fluorescence light. For this, microglial cells were seeded into white clear-bottom 96-well plates (30,000 cells/well). After becoming adherent, cells were stimulated as described above. For measurement, the medium was aspirated, and 200 μl/well of H_2_DCFDA-solution (stock 50 mM 1:1000 in 10 mM HEPES/CaCl_2_) was added, and cells were incubated for 20 min at 37° C. Thereafter, cells were carefully washed twice with an HEPES/CaCl_2_ solution. Measurement of intracellular ROS levels was performed at 485 nm excitation and 535 nm emission using a TECAN Infinite 200 Plate reader (Tecan, Switzerland).

### Lactate Production Measurement

Supernatants from microglial cultures were used to measure lactate production by sequential enzymatic reactions [according to Lin et al. (23)]. Briefly, lactate is converted by lactate oxidase (LO; #L0638, Sigma-Aldrich) to pyruvate and H_2_O_2_. In a second reaction, the chromogenic substrate ABTS (#A1888, Sigma-Aldrich) is converted to a colored dye, catalyzed by horseradish peroxidase (HRP; #77332, Sigma-Aldrich) in the presence of H_2_O_2_ and measured at 405 nm. Lactate levels were normalized to the total protein concentration of each sample.

### Statistical Analysis

Statistical analysis was carried out using SigmaPlot Software Version 13.0 Build 13.0.0.83 (Systat Software GmbH, Erkrath, Germany). Data are presented as scatter dot plots showing means ± SEM. Comparison between experimental groups and conditions were analyzed with two-way analysis of variance in all cases for the factors “Group” (three different age groups) and “State,” which consists of US (unstimulated), UP (unprimed), ULP (ultra-low primed), and HP (high primed). In all cases, interaction between both factors were analyzed. *Post-hoc* comparisons were made with the Holm–Sidak test. Differences were considered significant when *p* < 0.05.

## Results

### Effect of Priming With Low and High LPS Doses on Cytokine and iNOS Levels in Newborn, Adult, and Aged Microglia

Gene expression and protein levels of pro-inflammatory cytokines TNF-α, IL-6, and IL-1β were evaluated after repeated LPS stimulation in newborn, mature, and aged microglia and compared with related unstimulated (US) and unprimed (UP) microglial cells. As expected, US microglia expressed low cytokine levels (Figures 1B–E, *p* < 0.05). A single challenge with 100 ng/ml LPS (UP) led to a marked increase in levels of TNF- α, IL-6, and IL-1β in microglia in all groups (*p* < 0.05). Microglial priming with the ultra-low LPS dose (ULP) followed by a standard fixed LPS dose 5 days later further increased levels of TNF-α (Figures 1B,C), IL-6 (Figures 1D,E), and IL-1β (Figure 1F) in newborn microglia, indicating trained immunity (*p* < 0.05). No such effect was observed in mature and aged microglia for TNF-α and IL-6 after stimulation, whereas IL-1β was increased in microglia obtained from adult mice (Figure 1F, *p* < 0.05). In addition, expression of MMP-9, an important activation marker for microglia, was upregulated after low-dose LPS priming in newborn but not in mature microglia (Supplementary Figure 4E, *p* < 0.05). These results may suggest that induction of trained immunity leads to a pro-inflammatory phenotype mainly in newborn microglia.

In contrast, microglial priming with LPS at high doses (HP) led to a robust downregulation of pro-inflammatory cytokines in all groups 5 days after renewed challenge with LPS 100 ng/ml, indicating induced immune tolerance (*p* < 0.05). A similar effect was observed for mRNA expression of iNOS with newborn microglia displaying both trained immunity after priming with ultra-low LPS doses and tolerance after priming with high LPS concentrations (Figure 1G, *p* < 0.05). Mature and aged microglia did not show a significant response at either ultra-low or high LPS doses as compared with the unprimed group.

**Figure 2:**
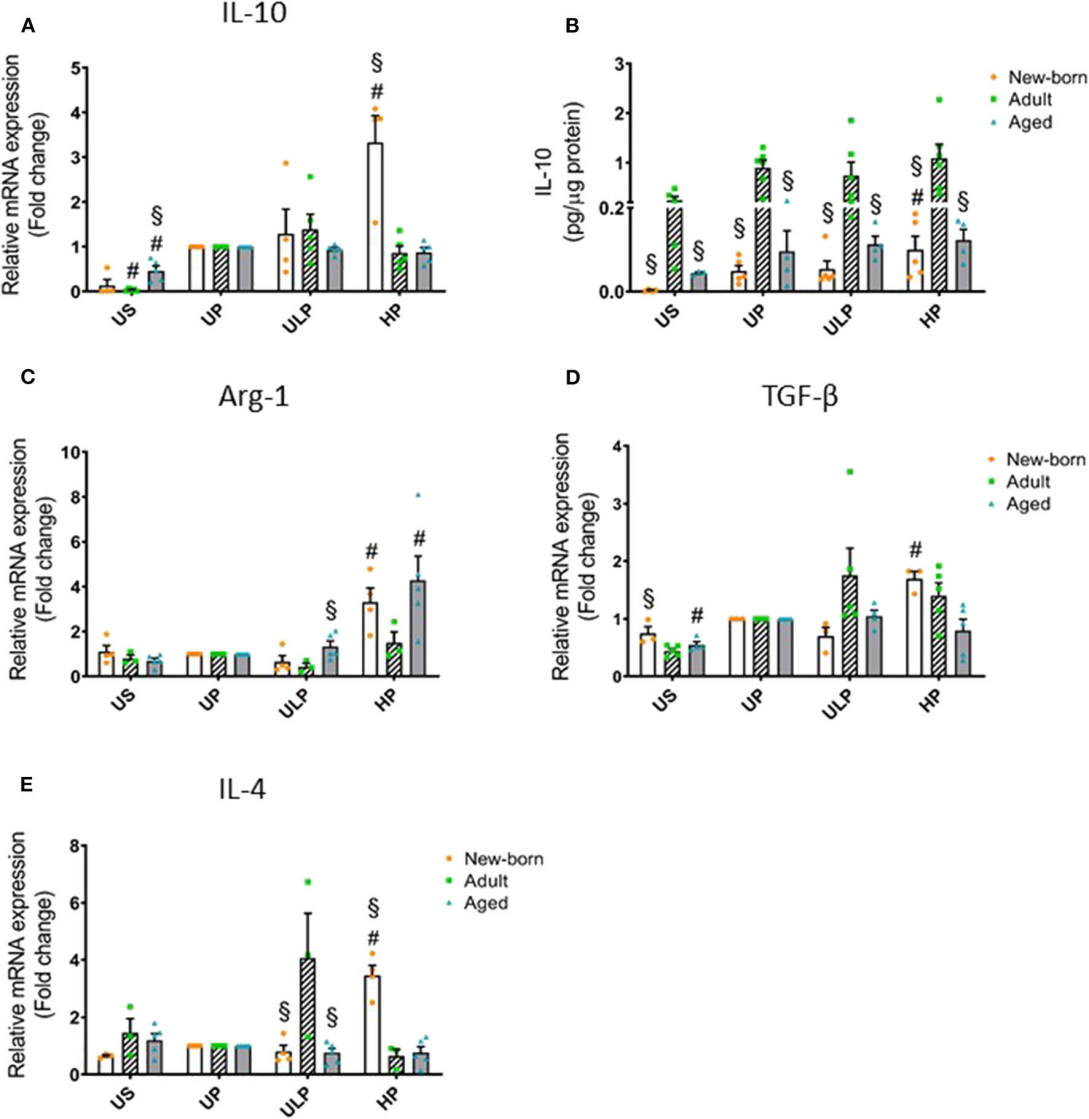
Effect of maturation and priming with low and high LPS dosages on anti-inflammatory responses of murine brain microglia. Microglia isolated from newborn (orange dots, open columns), adult (green dots, hatched columns), and aged (blue dots, gray columns) mice were primed initially by ultra-low (ULP, 1 fg/ml) or high (HP, 100 ng/ml) doses of LPS, followed by a second stimulation (day 6) with 100 ng/ml LPS. The data were normalized and compared to unprimed microglia (UP Group without any stimulation at day 1 with stimulation at day 6 with a fixed dose of LPS 100 ng/ml). Unstimulated microglia served as negative control (US Group without LPS stimulation neither at day 1 nor at day 6). RNA samples (6 h) and supernatants (24 h) were collected after the 2nd stimulation and analyzed for gene expression of IL-10 (**A**, *n* = 4–5), Arg-1 (**C**, *n* = 3–5), TGF-β (**D**, *n* = 3–5), and IL-4 (**E**, *n* = 3–5) (unprimed cells assigned as 1.0) and cytokine production of IL-10 (**B**, *n* = 5–6) by ELISA (normalized to total protein concentration). Data shown in scatter dot plots represent means + SEM, ^#^*p* < 0.05 vs. unprimed conditions within each age group (naïve or mature), ^§^*p* < 0.05 vs. adult microglia within each stimulation condition (US, UP, ULP, HP).

In order to determine whether induction of trained immunity or tolerance shifts the microglial phenotype to an anti-inflammatory state, we assessed microglial gene expression and protein level of anti-inflammatory IL-10 as well as of the immunosuppressive mediators Arg-1, TGF-β, and IL-4. Strikingly, IL-10 release was manyfold enhanced in supernatants of adult microglia under unstimulated conditions and after stimulation, irrespective of the stimulation procedure (Figure 2B, *p* < 0.05). Furthermore, increased gene expression and production of IL-10 after high-dose LPS priming appeared in newborn microglia (Figures 2A,B, *p* < 0.05). In addition priming with ultra-low LPS doses had no effect on levels of IL-10 and Arg-1 in microglia of all age groups under consideration (Figure 2). Priming with a high LPS dose increased gene expression of Arg-1, TGF-β, and IL-4 in newborn microglia as well as in aged microglia for Arg-1, but not in adult microglia (Figures 2C–E, *p* < 0.05). Furthermore, MSR1 was upregulated in newborn and adult microglia (Supplementary Figure 4F, *p* < 0.05). This indicates that induction of tolerance in naïve and mature microglia is associated with an anti-inflammatory phenotype.

Repetitive stimulation with low and high LPS dosages for a period of 3 days induced similar dose- and maturation-dependent microglial responses for TNF-α and IL-6 (Supplementary Figure 1, *p* < 0.05) as shown in response to one-time priming. In contrast, a single stimulation with an ultra-low LPS dose did not induce any response, whereas stimulation with high-dose LPS induced the anticipated marked pro-inflammatory response (Supplementary Figure 2, *p* < 0.05). These response patterns appeared irrespective of the mode of stimulation (e.g., one-time or repetitive stimulation), and cell viability of naïve and mature microglia appeared to be similar (Supplementary Figure 3). Furthermore, exploration of molecular mechanisms underlying the demonstrated microglial training effects revealed that priming with low LPS doses is induced by increased TLR4/MyD88/IRAK-4 activation only in naïve microglia (*p* < 0.05), whereas immune tolerance is mediated by reduced TLR4/MyD88/IRAK-4 activation in both naïve and mature microglia (Supplementary Figure 4, *p* < 0.05).

### Effect of Priming With Low and High LPS Dosages on ROS, BDNF, and Metabolic Indicators in Newborn, Adult, and Aged Microglia

**Figure 3:**
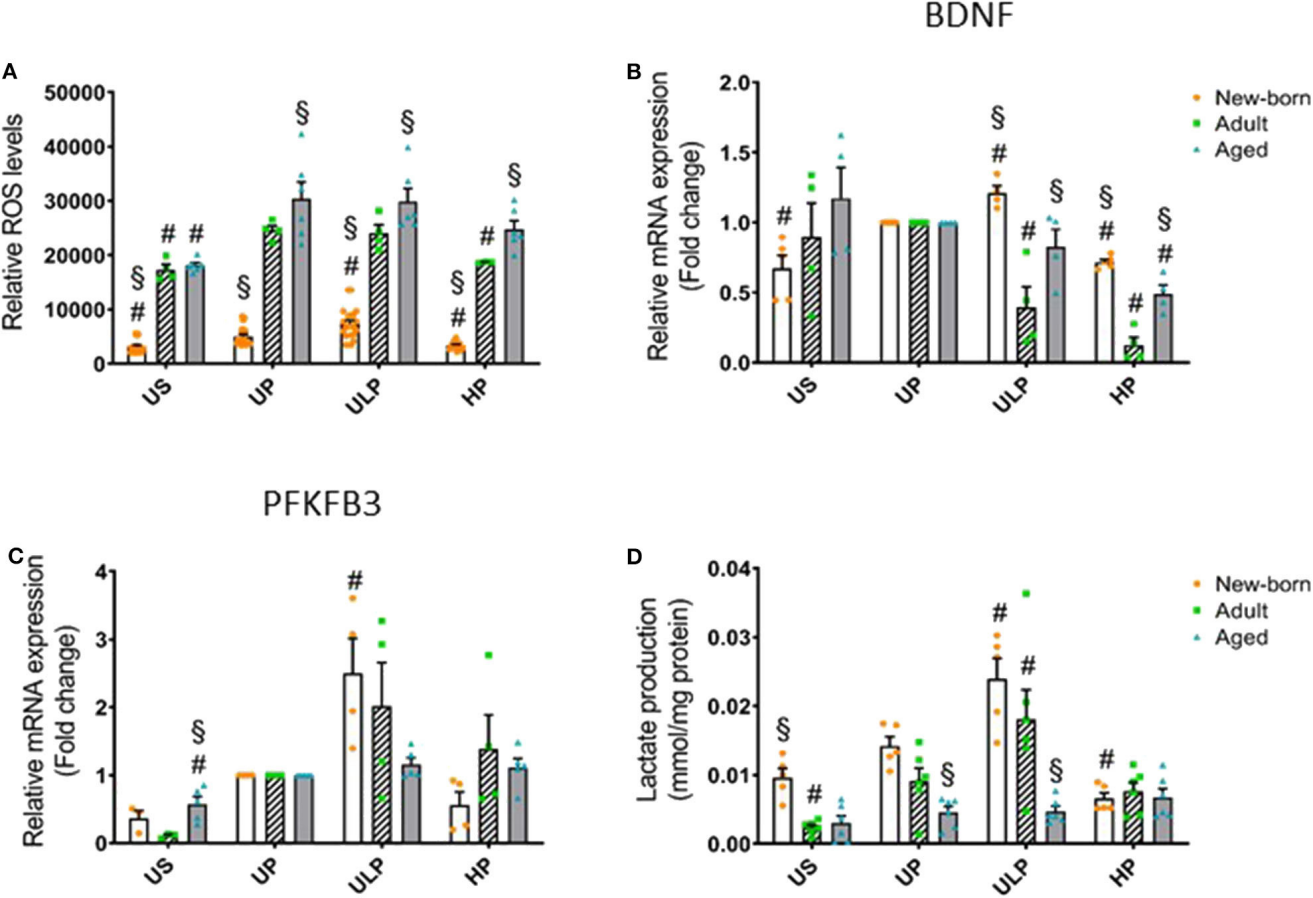
Effect of maturation and priming with low and high LPS dosages on ROS production, BDNF, and metabolic rewiring of murine brain microglia. Microglia isolated from newborn (orange dots, open columns), adult (green dots, hatched columns), and aged (blue dots, gray columns) mice were primed initially by ultra-low (ULP, 1 fg/ml) or high (HP, 100 ng/ml) doses of LPS, followed by a second stimulation (day 6) with 100 ng/ml LPS. The data were normalized and compared to unprimed microglia (UP Group without any stimulation at day 1 with stimulation at day 6 with fixed dose of LPS 100 ng/ml). Unstimulated microglia served as negative control (US Group without LPS stimulation neither at day 1 nor at day 6). RNA samples (6 h) and supernatant (24 h) were collected after the 2nd stimulation and analyzed for ROS production (**A**, *n* = 3–8, repeated measurements) and gene expression of BDNF (**B**, *n* = 4–5), and PFKFB3 (**C**, *n* = 4–5) (unprimed cells assigned as 1.0) as well as lactate concentration in supernatant (**D**, *n* = 5–6). Data shown in scatter dot plots represent means + SEM, ^#^*p* < 0.05 vs. unprimed conditions within each age group (naïve or mature), ^§^*p* < 0.05 vs. adult microglia within each stimulation condition (US, UP, ULP, HP).

Considering that reactive oxygen species (ROS) represent important executing molecules of innate immunity, we evaluated the impact of maturation and LPS dose on ROS production in microglia derived from newborn, adult, and aged mice brains. As shown in Figure 3A, unstimulated newborn naïve microglia showed distinctly lower ROS levels as compared with adult and aged microglia. Single stimulation with LPS significantly increased ROS levels in all microglia populations (Figure 3A, *p* < 0.05). ROS levels remained reduced in newborn microglia, whereas aged microglia exhibited increased ROS levels compared with adult microglia. Priming with ultra-low LPS doses followed by a second challenge 5 days later (day 6) further increased ROS levels in newborn (*p* < 0.05) but not in mature microglial cells. This response further supports induction of trained innate immunity as an exclusive feature of newborn microglia. In contrast, priming with high-dose LPS reduced ROS levels only in newborn and adult cell populations (*p* < 0.05), supporting induction of a tolerance state.

Given the widespread functional role of brain-derived neurotrophic factor (BNDF) released by microglia in physiological processes involved in learning and memory (24) and also in pathological events such as neuronal disinhibition of the intrinsic inhibitory system, causing neuropathic pain after peripheral nerve injury (25, 26), we evaluated the impact of the developmental state on gene regulation of microglial BNDF after repeated challenge with LPS. Priming with an ultra-low LPS dose increased BNDF expression in newborn microglia but reduced it strongly in adult microglia (Figure 3B, *p* < 0.05). Furthermore, priming of newborn microglia with high-dose LPS led to a diminished response after the second LPS stimulus compared with the unprimed state (*p* < 0.05). In mature microglial cells, high-dose priming with LPS further decreased BDNF expression (*p* < 0.05). Interestingly, after both ultra-low and high priming doses of LPS, BDNF expression levels appeared significantly higher in newborn and aged microglia when compared with adult microglia (*p* < 0.05).

Considering that metabolic reprogramming has been identified to be a crucial step for the induction of trained immunity in peripheral innate immune cells (27), we assessed the expression of 6-phosphofructo-2-kinase/fructose-2,6-biphosphatase (PFKFB)3, a rate-limiting enzyme of glycolysis and lactate production, in newborn, adult, and aged microglia. As shown in Figure 3C, gene expression of the glycolytic enzyme PFKFB3 was slightly increased in unstimulated microglia obtained from aged mice (*p* < 0.05), but baseline lactate concentration was enhanced in the supernatant of newborn naïve microglia (Figure 3D, *p* < 0.05). Priming with the ultra-low LPS dose induced a significant increase in PFKFB3 gene expression in newborn microglia as well as enhanced lactate concentration (Figures 3C,D, *p* < 0.05). In contrast, priming with high-dose LPS significantly reduced lactate concentration in the supernatants of newborn naïve microglia cultures (Figure 3D, *p* < 0.05).

### Effect of Priming With Low and High β-glucan Dosages on Cytokine Levels in Newborn, Adult, and Aged Microglia

**Figure 4:**
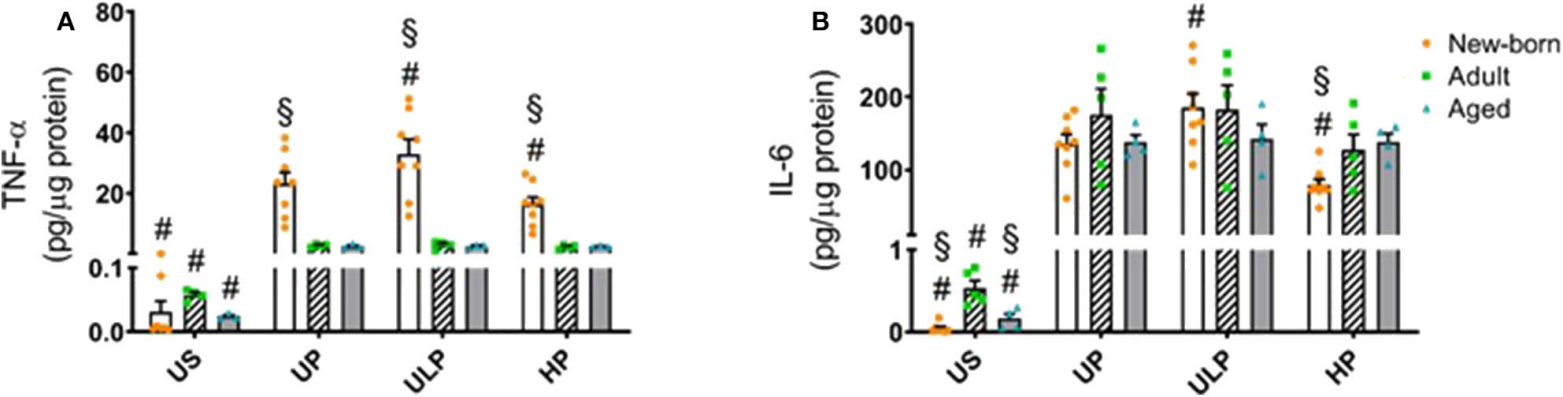
Effect of maturation and priming with low and high β-glucan dosages on proinflammatory responses of murine brain microglia. Microglia isolated from newborn (orange dots, open columns), adult (green dots, hatched columns), and aged (blue dots, gray columns) mice were primed initially by ultra-low (ULP, 100 fg/ml) or high (HP, 1 μg/ml) doses of β-glucan, followed by a second stimulation (day 6) with 100 ng/ml LPS. The data were normalized and compared to unprimed microglia (UP Group without any stimulation at day 1 with stimulation at day 6 with fixed dose of LPS 100 ng/ml). Unstimulated microglia served as negative control (US Group without β-glucan stimulation neither at day 1 nor at day 6). Supernatants were collected 24 h after the 2nd stimulation and analyzed for TNFα **(A)** (*n* = 4–8) and IL-6 **(B)** (*n* = 5–8) levels by ELISA (normalized to total protein concentration). Data shown in scatter dot plots represent means + SEM, ^#^*p* < 0.05 vs. unprimed conditions within each age group (naïve or mature), ^§^*p* < 0.05 vs. adult microglia within each stimulation condition (US, UP, ULP, HP).

In order to further characterize the impact of maturation on microglial response after repeated challenge with PAMPs, cells were primed with different doses of β-glucan, a fungal cell wall component from *Candida albicans*, followed by a stimulation with LPS (100 ng/ml) at day 6. Priming with ultra-low β*-glucan* doses further increased levels of TNF-α and IL-6, and high concentrations reduced release of both cytokines in newborn microglia (Figure 4, *p* < 0.05). In contrast, adult and aged microglia did not show a relevant response to β-glucan. These findings suggest a β-glucan-mediated dose-dependent induction of trained immunity and tolerance only in newborn microglia as previously shown for LPS (14, 15, 18).

## Discussion

Our study supports recent evidence indicating memory-like responses of brain microglia after repeated immune challenge (14, 15, 18). Corroborating our previous report (14, 15, 18), priming of neonatal microglia with low doses of LPS was found to increase release of pro-inflammatory cytokines after the second challenge, whereas priming the cells with high doses of LPS induces tolerance (i.e., decreased release of these cytokines). Collectively, these findings fit into the paradigm of pathogen dose-dependent training and tolerance responses recently developed for peripheral innate immune cells (28, 29).

In our present study, we show for the first time, that the patterns of adaptive responses in microglia are regulated by the developmental state of these cells. Moreover, independently of the mode of priming (one-time vs. repetitively) microglia consistently developed an immune response suggesting that microglial immune memory can play a role under widespread conditions of PAMP confrontation. Furthermore, our data reveal that the fungal cell wall constituent β-glucan also provokes development- and dose-dependent training and tolerance responses. Considering that β-glucan is recognized by a different receptor as LPS (Dectin-1 vs. TLR-4), these data provide evidence that similar microglial responses (increased levels of TNF and IL-6 after ULP) might arise after stimulation with compounds that mimic different types of infections and develop into trained immunity. Notably, a remarkable shift of the sensitivity of the affected microglial cells can be relevant. Whereas, LPS-induced training was observed at 1 fg/ml LPS, β-glucan provokes training at 100 fg/ml. Different effective concentrations of LPS and β-glucan might result from varying affinities of the TLR-4 and Dectin-1 receptors binding these two PAMPs. The high sensitivity of microglia to LPS might be a special characteristic of neonatal cells (30).

The observed training responses of microglia derived from neonatal mouse brains after priming with an ultra-low LPS dose raises the question of whether this challenge is of physiological relevance or may result from particular experimental conditions. Previous studies revealed that LPS is extraordinarily potent and able to stimulate host responses even in the femtomolar range corresponding to about 100 invading Gram-negative bacteria (31–33). Mechanistically, the LPS-induced immune cell activation is mediated by TLR4 (34, 35). Receptor activation requires binding of an individual LPS molecule (LPS monomer) to the MD-2/TLR4 dimeric receptor (36) and dimerization of the LPS.MD-2.TLR4 ternary complex (37). In turn, as few as 25 LPS MD-2.TLR4 complexes per cell can trigger measurable pro-inflammatory responses, implying very efficient oligomerization of these ternary complexes (38). Therefore, priming effects with ultra-low LPS doses are apparently potent enough to control intracellular training effects important for innate immune memory in neonatal microglia as we have shown previously (18). Mature microglia also responded to ultra-low LPS priming with increased expression of TLR4, MyD88, and IRAK-4 but exhibited no upregulation of the key transcription factor p65, suggesting a loss of pro-inflammatory-mediated activation via the canonical NF-κB pathway (Supplementary Figure 4) (39, 40). In contrast, priming with a high LPS dosage was accompanied by a suppression of the pro-inflammatory TLR4-IRAK-4-p65 axis, indicating induced immune tolerance (41, 42).

In contrast to the trained response pattern of neonatal microglia, microglia derived from mature mice brains did not exhibit a similar reinforced response after priming with ultra-low doses of LPS. These findings are in line with a previous study on primary microglia isolated from adult mice, indicating development of immune tolerance but not training, after stimulation with high doses of LPS (14). In our hands, microglia derived from aged mice showed a shift toward downregulation of pro-inflammatory cytokines and upregulation of anti-inflammatory mediators after priming with an ultra-low LPS dose. These findings are also in line with previous evidence showing that, with aging, sensome transcripts for endogenous ligand recognition are downregulated, whereas those involved in host defense are upregulated. Furthermore, aging was associated with an overall increase in the expression of microglial genes involved in neuroprotection (43).

Therefore, increased sensitivity of immature microglia toward low doses of microbial PAMPs and their downward turn during the maturation process appear to be a tightly regulated adaptive response of the innate immune cells in the brain to the microbial gut population, possibly associated with intense brain–gut interactions (44, 45). Specifically, recent studies have shown that microglial development proceeds in a stepwise manner with strong variations between different regions of the CNS, driven by transcriptional and epigenomic regulation and mediated by distinct pathways responsible for processing the relevant signals from the environment to balance their time-dependent role in neurogenesis with the regulation of immune responses (46, 47). Clearly, host microbiota represent a major environmental factor for microglial maturation and markedly modulates microglia properties. Therefore, microglial homeostasis after maturation is mainly regulated by microbiota-derived bacterial fermentation products and mediated by histone modifications (48). However, due to proper microbial gut colonization during the neonatal period, a microglial maturation spurt occurs, which is characterized by increased proliferation and enhanced expression of genes associated with inflammation and defense response (46, 49). Furthermore, several processes, including synaptogenesis, regulation of neurotransmitters and neurotrophic factors (50), blood–brain barrier tightness, and upregulated expression of tight junction proteins, among others, are mediated by microbiota–brain communication (51). In addition, it appears that early microglial confrontation with microbiota-derived TLR4 ligands is essential to prime brain immune competence not only for microbial challenges, but also for viral infections (52, 53).

Interestingly, priming with an ultra-low LPS dose did not induce an accompanying anti-inflammatory cytokine response as indicated by a missing IL-10 elevation in the three age groups studied (Figure 2). The rationale for these findings cannot be drawn from our results. Nevertheless, it can be speculated that microglia respond to priming with ultra-low LPS doses, such as leukocytes with non-resolving inflammatory adaptation, by removal of negative modulators, disallowing the development of anti-inflammatory tolerance and favoring the inflammatory monocyte adaptation (54).

Intriguingly, microglial immune tolerance resulting in a dampened pro-inflammatory response after repeated high-dose LPS challenge was similar in the different age states under consideration. Obviously, tight control of the neuro-inflammatory response over time appears to be an imperative prerequisite to preserve brain tissue from harm due to overwhelming inflammation. Therefore, the high dose-tolerance effects might mainly reflect a regulatory response resulting from IL-10 upregulation in immature microglia (18). This consideration derived from our data is in line with previous reports and may preclude an exhaustion of the machinery to produce pro-inflammatory cytokines [for review, see Lobo-Silva et al. (55)]. In contrast, microglial restorative activity at high-dose LPS was more pronounced in microglia derived from newborn mice, underscoring the inherent neuroprotective capacity of immature microglia. This specific response pattern of neonatal microglia might be relevant for the compensation of vulnerable effects of inflammation in the immature brain (56).

Importantly, expression of the PAMP receptor TLR-4, its associated protein MyD88, and the key downstream kinase IRAK-4 (which promotes phosphorylation of IRAK-1), which is essential for TLR-mediated NF-κB activation, were increased after priming with ultra-low LPS dose in naïve and mature microglia (Supplementary Figure 4). However, upregulation of p65, a key element for NF-kB activation, exhibited a pronounced training response only in primed newborn microglia. In contrast, priming with a high dose was accompanied by downregulation of the NF-kB in both age groups, indicating the molecular basis of induced immune tolerance in microglia. Strikingly, sensitization of NF-kB by priming with a lower LPS dosage (herein seen in naïve microglia) and tolerance development by a higher LPS dose (observed in naïve and mature microglia) has been recently observed by single-cell analysis and assigned to a dose-sensing autoregulatory loop via IL-1R-associated kinase 1 (IRAK1) (57).

Current research identifies metabolic reprogramming as a key hallmark of innate immunity along with immune cell activation. It is characterized by a fine-tuned pattern of metabolic conditions that become rewired after repeated immunological challenges, probably via epigenetic modifications (58, 59). We find here that trained immunity driven by repeated LPS challenges in newborn microglia is associated with a shift toward aerobic glycolysis as the dominating immunometabolic process herein. PFKFB3-driven macrophage glycolytic metabolism is found to be a crucial component of the innate immune response (60). Induction of the glycolytic activator PFKFB3 and increased lactate production suggest a possible TCA-cycle remodeling, probably by itaconate, as previously described elsewhere (61).

Release of reactive oxygen species was several times higher by mature and aged microglia compared to microglia derived from newborn pups exhibiting a dose-dependent response after priming (e.g., trained immunity and immune tolerance). These data suggest that the immature brain appears to be especially vulnerable against oxidative stress. There is compelling evidence that high concentrations of unsaturated fatty acids, a high rate of oxygen consumption, low concentrations of antioxidants, and increased availability of “free” redox-active iron might be responsible for this effect (62–64).

The *in vitro* approach used in our study has several advantages compared to *in vivo* approaches and permits the identification of basic molecular mechanisms involved in microglial priming under specific experimental conditions, avoiding the effect of paracrine factors or complex environmental effects. We used primary cells instead of immortalized cell lines, which more closely resemble cellular entities in the naïve tissue (21, 65). The presented data reveal no relevant difference in cell viability and protein concentration between age groups and experimental procedures, suggesting comparable cellular responses between naïve and mature microglial cells (Supplementary Figure 3).

On the other hand, we have to consider several limitations inherent to the microglial cell culture approach used. First of all, it is well-known that microglial cells react to the cell culture environment with the consequence that *in vitro* studies may not faithfully reflect properties of microglia under physiological conditions (8). Furthermore, the translational value of data obtained from microglial cell culture studies is somehow limited due to the complex function of microglial cells in brain tissues. In particular, microglia, considered to be the prototypic tissue-resident macrophage-like innate immune cells of the CNS, are multifunctional cells that interact with numerous other cells in the CNS, including neurons, astrocytes, and oligodendrocytes (66). *In vitro* studies using cell cultures with isolated microglia preclude *per se* these essential interactions given in brain tissue. Therefore, response patterns of microglia are curtailed inevitably but deliver important reference points for subsequent and productive translational research. Recently, seminal contributions to preclinical neurological research have been published that identify immune memory in the brain as an important modifier of neuropathology under *in vivo* conditions (15). Specifically, peripherally applied inflammatory stimuli induce acute immune training and tolerance in the brain and lead to differential epigenetic reprogramming in microglia that persists for several months. Strikingly, in a mouse model of Alzheimer's pathology, immune training exacerbated cerebral β-amyloidosis, and immune tolerance alleviated it (16). Similarly, peripheral immune stimulation modified pathological features after stroke. This evidence impressively demonstrates the role of microglial immune memory in pathological hallmarks of neurological disease in mouse models, which is obviously consistent with certain clinical observations in patients (16).

Another source of limitations may result from methodical reasons. For example, in this study, we used exclusively male adult and aged mice for microglial cell isolation in order to compare it with previous studies in male mice in our lab, involving aging and senescence of microglia (20).

Consequently, we have to consider that this approach may restrict the spectrum of response patterns given under (patho)physiological conditions. Although there are still no data available, analysis of differential gender-associated microglia immune memory responses may be relevant. In support of this, a recent study by Villa et al. (67) finds gender-associated differences at the transcriptome level in adult murine microglia (67). Furthermore, female microglia showed a neuroprotective phenotype, which was independent from hormonal cues. Findings concerning gender-specific innate immune memory responses have already been described for peripheral blood mononuclear cells derived from humans after Bacillus Calmette-Guerin (BCG) vaccination. While BCG vaccination enhanced cytokine responses to restimulation, it reduced systemic inflammation. This effect was much stronger in men than in women. Hence, the capacity of BCG to enhance microbial responsiveness while dampening systemic inflammation may be clinically relevant for potential therapeutic applications (68).

In summary, we find a differential response to repeated PAMP challenges between naïve microglia obtained from newborn murine brain and adult as well as aged microglia. Whereas, naïve microglia appear to be prone for an orchestrated pro-inflammatory response in a dose-dependent manner, resulting in trained immunity after priming with ultra-low dose LPS, mature and aged microglia did not show trained immunity, possibly reflecting the need for precluding excessive damage of the central nervous system after recurrent systemic inflammation.

## Data Availability Statement

The raw data supporting the conclusions of this article will be made available by the authors, without undue reservation.

## Ethics Statement

The studies involving animals were reviewed and approved by the Thuringian State Office for Food Safety and Consumer Protection.

## Author Contributions

TL, MS, RB, RW, and CS contributed to the conception and design of the study. DW provided the *C. albicans* derived β-glucan. TL and MS performed the experiments. TL, RB, and MS performed the statistical analyses. CS, RW, and RB wrote the manuscript. OW, HH, and MB critically discussed the manuscript. All authors contributed to manuscript revision, read, and approved the submitted version.

## Conflict of Interest

The authors declare that the research was conducted in the absence of any commercial or financial relationships that could be construed as a potential conflict of interest.
