# Supplementary material for: Memory-Like Responses of Brain Microglia Are Controlled by Developmental State and Pathogen Dose
Source: Front Immunol. 2020 Sep 25;11:546415. doi: 10.3389/fimmu.2020.546415 (PMC7546897; doi:10.3389/fimmu.2020.546415)
Supplement: Supplementary file 1 [file Table_1.docx]

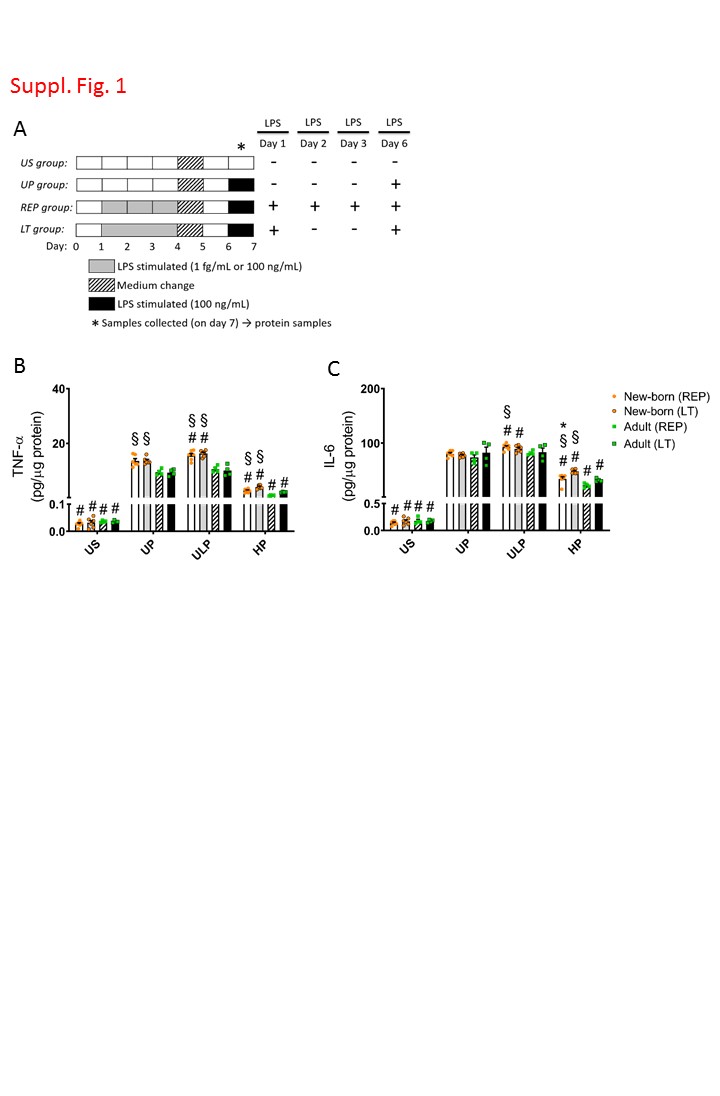


Supplementary Figure S1: Effects of maturation, priming with low and high LPS dosages, repetitive or long-term administration and final LPS stimulation on pro-inflammatory responses of murine brain microglia.

Microglia isolated from newborn (orange dots) and adult (green dots) were (i) repetitively primed by ultra-low (ULP, 1 fg/ml) or high (HP, 100 ng/ml) doses of LPS, once a day for 3 days (repetitive (REP) group, open columns, newborn; hatched columns, adult), (ii) primed for 3 days (long-term (LT) group, gray columns- newborn; black columns, adult), followed by challenge with 100 ng/ml LPS on day 6. (A) Stimulation diagram. The data were normalized and compared to unprimed (UP) microglia. Data obtained from unstimulated (US) microglia were used as negative control. Supernatants were collected on day 7. Levels of TNF-α (B, n=4-6) and IL-6 (C, n=4-6) were determined by ELISA (values are then normalized to the total protein concentration). Data shown in scatter dot plots represent means + SEM, ^#^ p <0.05 vs. unprimed conditions within each age group, ^§^ p <0.05 vs. adult microglia within each stimulation condition, and * p <0.05 vs. long-term (LT) stimulation within the responding aging group.


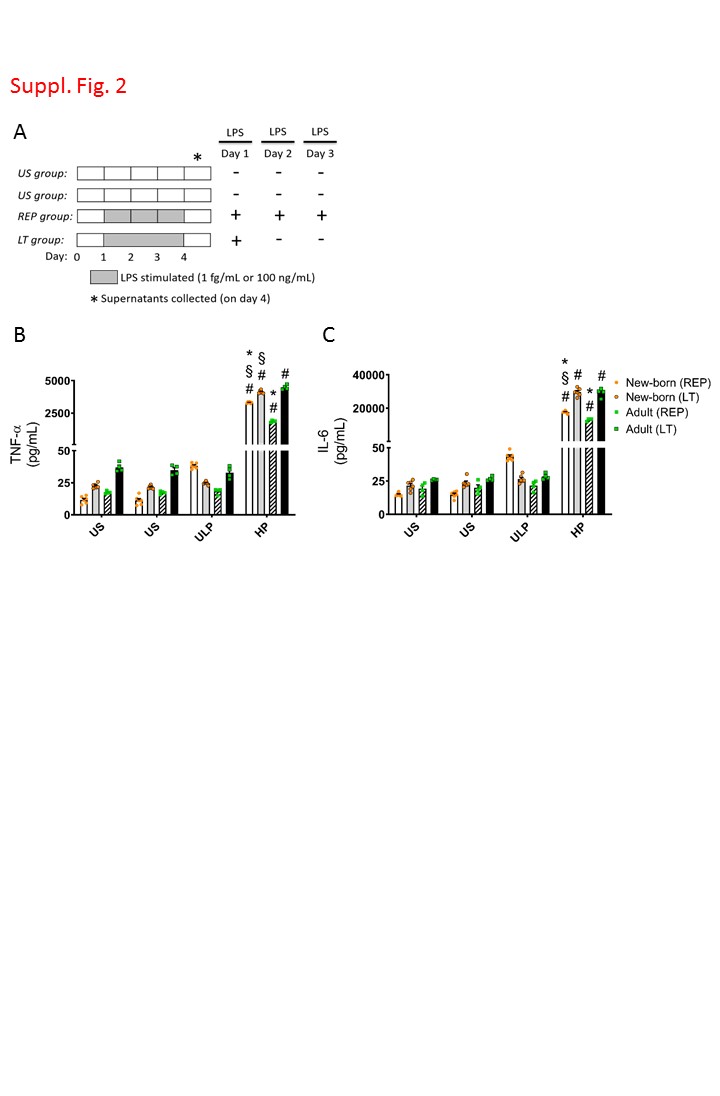


Supplementary Figure S2: Effects of maturation and repetitive and long-term stimulation on pro-inflammatory responses of murine brain microglia.

Microglia isolated from newborn (orange dots) and adult (green dots) mice were (i) primed by ultra-low (ULP, 1 fg/ml) or high (HP, 100 ng/ml) doses of LPS, repetitively once a day for 3 days (repetitive (REP) group, open columns, newborn; hatched columns, adult), (ii) primed onetime for 3 days (long-term (LT) group, gray columns, newborn; black columns, adult). (A) Stimulation diagram. Supernatants were collected on day 4. Measurement of cytokine production for TNF-α (B) (n=4-6) and IL-6 (C) (n=4-6) were performed by ELISA. Data obtained from unstimulated (US) microglia were used as control. Data shown in scatter dot plots represent means + SEM, ^#^ p <0.05 vs. unstimulated conditions within each age group, ^§^ p <0.05 vs. adult microglia within each stimulation condition, and * p <0.05 vs. long-term (LT) stimulation within the responding aging group.


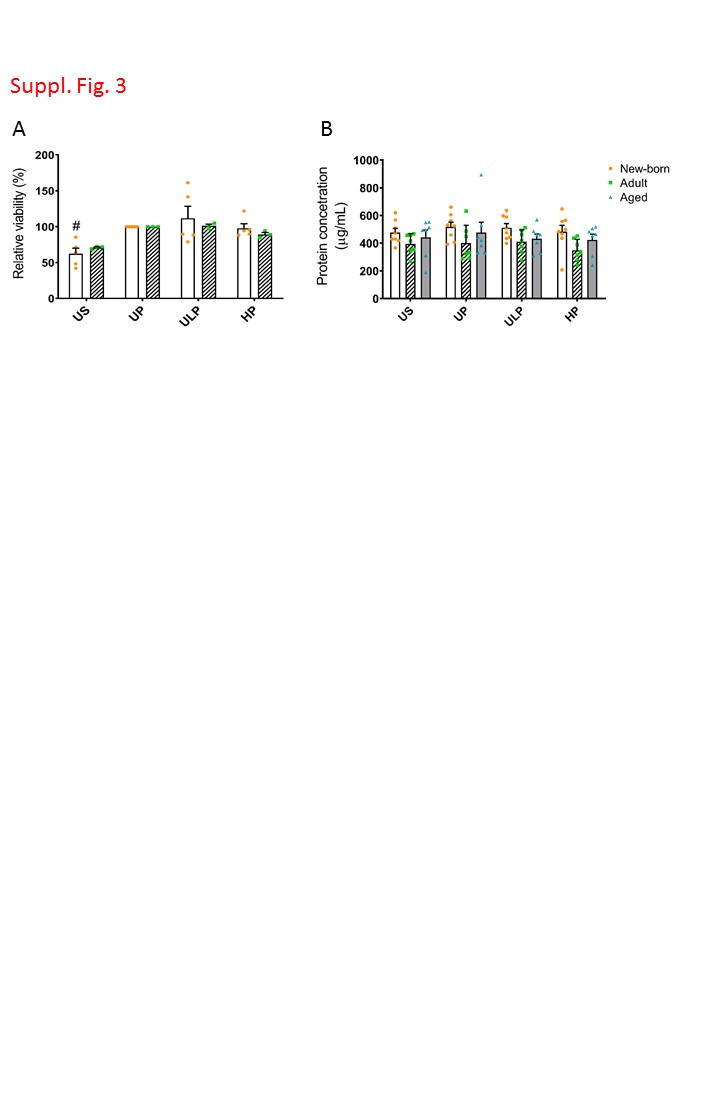


Supplementary Figure S3: Cell viability and protein concentration in primed microglial cells.

Microglia isolated from newborn (orange dots, open columns), adult (green dots, hatched columns) and aged (blue dots, gray columns) mice were primed initially by ultra-low (ULP, 1 fg/ml) or high (HP, 100 ng/ml) doses of LPS, followed by a second stimulation (day 6) with 100 ng/ml LPS. Cell viability was measured using the MTT assay (A, n=3-5; unprimed cells assigned as 100%), whereas the total protein concentration was analyzed using Pierce™ 660 nm Protein Assay Kit (B, n=7-8). Data are shown as scatter dot plots as means + SEM, ^#^ p <0.05 vs. unprimed conditions within each age group.


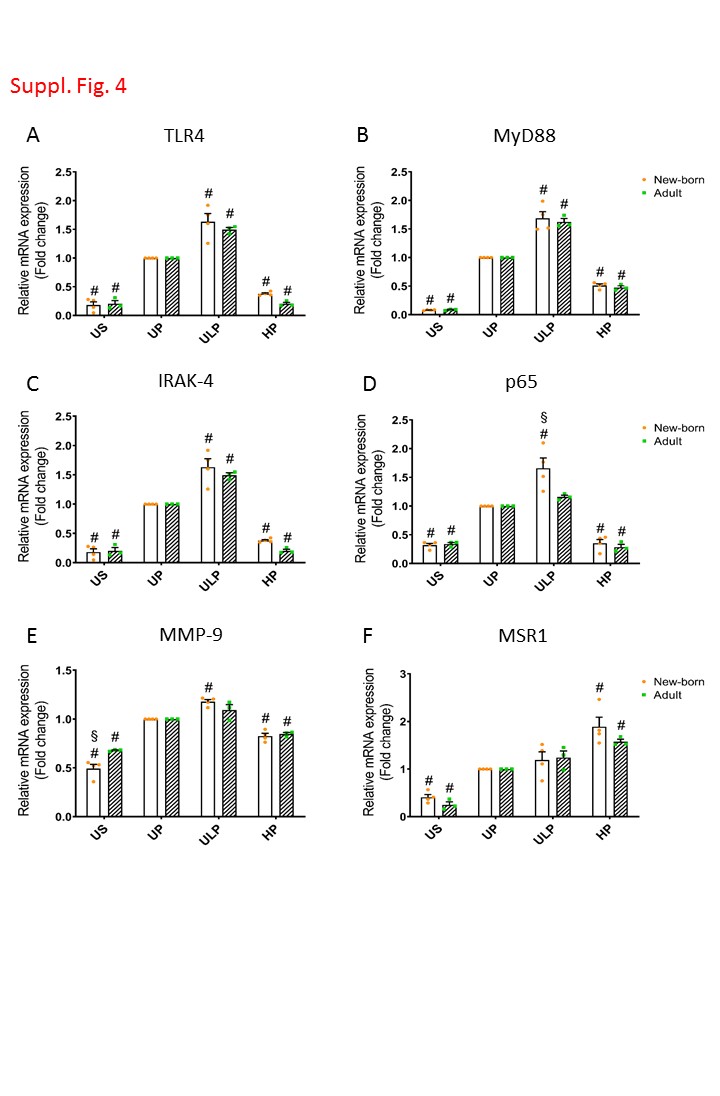


Supplementary Figure S4: Effects of maturation and priming with low and high LPS dosages on molecular mechanism of pro-inflammatory and anti-inflammatory responses of murine brain microglia.

Microglia isolated from newborn (orange dots, open columns) and adult (green dots, hatched columns) mice were primed initially by ultra-low (ULP, 1 fg/ml) or high (HP, 100 ng/ml) doses of LPS, followed by a second stimulation (day 6) with 100 ng/ml LPS. The data are normalized and compared to unprimed microglia (UP). Unstimulated microglia served as negative control (US). RNA samples (6h) were collected after the 2nd stimulation and analyzed for gene expression of TLR4 (A, n=3-4), MyD88 (B, n=3-4), IRAK-4 (C, n=3-4), p65 (D, n=3-4), MMP-9 (E, n=3-4) and MSR1 (F, n=3-4) (unprimed cells assigned as 1.0). Data shown in scatter dot plots represent means + SEM, ^#^ p <0.05 vs. unprimed conditions within each age group, ^§^ p <0.05 vs. adult microglia within each stimulation condition (US, UP, ULP, HP).
